# Lactic acid regulates antitumor immunity in canine invasive urothelial carcinoma

**DOI:** 10.1371/journal.pone.0332825

**Published:** 2025-09-18

**Authors:** Taiki Kato, Nao Okauchi, Tomoki Motegi, Masashi Sakurai, Shingo Maeda, Daiki Kato, Takayuki Nakagawa, Kazuyuki Uchida, Takuya Mizuno, Masaya Igase

**Affiliations:** 1 Laboratory of Molecular Diagnostics and Therapeutics, Joint Faculty of Veterinary Medicine, Yamaguchi University, Yamaguchi, Japan; 2 Section of Computational Biomedicine, Boston University Chobanian & Avedisian School of Medicine, Boston, Massachusetts, United States of America; 3 Laboratory of Veterinary Pathology, Joint Faculty of Veterinary Medicine, Yamaguchi University, Yamaguchi, Japan; 4 Laboratory of Veterinary Clinical Pathobiology, Graduate School of Agriculture and Life Sciences, The University of Tokyo, Tokyo, Japan; 5 Laboratory of Veterinary Surgery, Graduate School of Agriculture and Life Sciences, The University of Tokyo, Tokyo, Japan; 6 Laboratory of Veterinary Pathology, Graduate School of Agricultural and Life Sciences, The University of Tokyo, Tokyo, Japan; 7 Research Institute for Cell Design Medical Science, Yamaguchi University, Ube, Japan; 8 Japan Small Animal Cancer Center, Public Interest Incorporated Foundation Japan Small Animal Medical Center, Tokorozawa, Saitama, Japan; King Faisal Specialist Hospital and Research Center, SAUDI ARABIA

## Abstract

Canine invasive urothelial carcinoma (iUC) is a fatal malignant neoplasm that closely resembles human muscle-invasive bladder cancer in terms of histopathological features, molecular alterations, and clinical behavior. These similarities suggest that canine iUC represents a valuable spontaneous model for studying human bladder cancer. Tumor microenvironment (TME) plays a crucial role in tumor progression. Tumor-derived lactic acid has been implicated in the suppression of antitumor immunity and the promotion of tumor growth by altering the metabolic status of immune cells within the TME. However, the interaction between tumor metabolism and immune cells in the TME remains unclear in dogs. This study reanalyzed previously reported RNA-seq data to investigate the mechanisms underlying enhanced glycolysis in canine iUC. ERBB2 overexpression was found to induce AKT phosphorylation and increase extracellular lactic acid levels *in vitro*, activating the ERBB2-AKT-glycolysis axis and upregulating monocarboxylate transporter 4 (MCT4). MCT4 knockdown by RNA interference or pharmacological inhibition with diclofenac reduced lactic acid levels in the culture supernatant. Furthermore, MCT4 expression in canine iUC tissues was positively correlated with infiltrating regulatory T cell (Treg) counts. Functional studies revealed that lactic acid promoted Treg differentiation and suppressed IFN-γ production by effector T cells. These findings indicate that MCT4 mediates lactic acid efflux from glycolytic tumor cells, contributing to the suppression of antitumor immunity. Targeting tumor metabolism through MCT4 inhibition may represent a promising therapeutic strategy for canine iUC. Therefore, insights from the metabolic and immunological landscape of canine iUC may inform the development of translational therapies for both veterinary and human oncology.

## Introduction

Invasive urothelial carcinoma (iUC) is the most common malignant tumor of the canine urinary bladder, with the majority of cases already invaded the muscle layers at the time of diagnosis [1]. iUC has a poor prognosis and a high metastatic rate to regional lymph nodes and other organs, such as lungs, bones, and liver [2]. There are several treatment options, including surgery, chemotherapy, and radiation therapy. Piroxicam, the non-steroidal anti-inflammatory drug (NSAID), was primarily chosen for antitumor effects in iUC compared to other tumors [3].

The tumor microenvironment (TME) comprises not only tumor cells but also various cellular components, including blood vessels, stroma, and immune cells, where nutrient, oxygen, and pH levels are significantly lower than in other body organs. Cancer cells modify their metabolic patterns to adapt to the hypoxic TME, a process known as “metabolic reprogramming” [4]. In hypoxic tumor cells, glycolysis is highly active rather than oxidative phosphorylation, resulting in glucose depletion and substantial lactic acid accumulation in the TME [5]. Furthermore, tumor-derived lactic acid has been shown to promote tumor growth by suppressing antitumor immunity via inhibition of glycolytic metabolism in T cells [6]. Regulatory T (Treg) cells maintain their suppressive function under high lactic acid conditions in the TME. In contrast, effector T cells exhibit impaired proliferation and reduced cytokine production in response to tumors [6–8]. However, targeting metabolism in the TME remains poorly understood in canine cancers.

In cancer cells, glucose is converted to pyruvate, and most pyruvate undergoes aerobic glycolysis rather than oxidative phosphorylation, resulting in substantial lactic acid production. Consequently, pyruvate is utilized for adenosine triphosphate production, leading to lactic acid accumulation. Monocarboxylate transporter 4 (MCT4, also known as SLC16A3), a member of the monocarboxylate transporter family, plays a crucial role in lactic acid efflux from cells [9], contributing to the acidic TME. Notably, MCT4 is strongly expressed in highly glycolytic cells, such as tumors [10]. Higher MCT4 expression was associated with poor prognosis in human lung adenocarcinoma [11], breast cancer [12], and iUCs [13]. We previously revealed that MCT4 protein was mostly found in urothelial carcinoma and lung adenocarcinoma in dogs [14].

Oncogenic mutations are suggested to influence the metabolic profile of the TME by promoting metabolic reprogramming in cancer cells [15]. The human epidermal growth factor receptor 2 (HER2/ERBB2) is a transmembrane tyrosine kinase receptor involved in the regulation and maintenance of cellular functions, including cell proliferation, migration, and differentiation [16,17]. ERBB2 signaling is typically initiated through homo- or heterodimerization with other ERBB family members, including EGFR, ERBB3, and ERBB4. ERBB2 dimerization has been shown to activate signaling molecules that regulate tumor metabolism [18–20]. A previous study demonstrated that ERBB2 enhanced tumor cell growth and glycolysis through LDHA upregulation [20]. In canine iUC, ERBB2 overexpression and gene amplification were observed in 60% and 35% of cases, respectively [21]. However, the relationship between ERBB2 signaling and tumor metabolism in dogs remains poorly understood. Therefore, further investigation of molecular mechanisms, including metabolism in canine iUC, is necessary to develop novel therapeutic approaches.

In addition to its clinical relevance in veterinary medicine, canine iUC shares multiple histopathological, molecular, and clinical characteristics with human muscle-invasive bladder cancer, including aggressive behavior, metastatic patterns, and molecular alterations such as ERBB2 overexpression [22,23]. Owing to these similarities, spontaneous canine iUC has been recognized as a valuable comparative and translational model for investigating the pathogenesis of human bladder cancer and for evaluating novel therapeutic strategies. Therefore, exploring tumor metabolism and immune responses in canine iUC not only advances veterinary oncology but also provides important insights relevant to human cancer research.

We focused on the impact of lactic acid on canine antitumor immunity. We investigated extracellular lactic acid levels, which was evaluated in ERBB2-overexpressing and MCT4-knockdown iUC cell lines. Moreover, we assessed the effect of lactic acid on canine effector T cell function and *in vitro* Treg differentiation. Finally, we evaluated the cytotoxicity and extracellular lactic acid levels suppression of an MCT4 inhibitor in canine iUC cells.

## Materials and methods

### Statement of cell line validation and culture

The canine invasive urothelial carcinoma (iUC) cell lines Sora, Nene, and Love [24] were cultured in complete RPMI medium (R10), comprising RPMI-1640 medium supplemented with 10% fetal bovine serum (FBS), 100 units/mL penicillin-streptomycin, and 55 μM 2-mercaptoethanol. These cells were maintained at 37°C in a humidified incubator with 5% CO_2_. For viral packaging, PLAT-gp and HEK293T cells were cultured in complete medium (D10) consisting of high-glucose Dulbecco’s Modified Eagle Medium supplemented with 10% FBS, 100 units/mL penicillin-streptomycin, and 55 μM 2-mercaptoethanol. All canine iUC cell lines were kindly provided by Dr. Takayuki Nakagawa (University of Tokyo). The absence of mycoplasma contamination in all cell lines was confirmed using the e-MycoTM Plus Mycoplasma PCR Detection Kit (iNtRON Bio, Burlington, MA).

### Ethics approval and consent to participate

The Ethics Review Board of Yamaguchi University approved this study using our laboratory’s tissue archives. All owners provided written informed consent for the use of each FFPE tissue specimen. The experiment involving canine primary blood samples was approved by the Yamaguchi University Ethics Committee (Approval No. 580) and conducted in accordance with the Yamaguchi University Guidelines for Animal Care and Use. We confirm that all our methods comply with our university’s guidelines for animal experimentation and that our work is reported following the ARRIVE guidelines.

### Specimens

We used 7 FFPE normal tissue samples and 10 FFPE tumor tissue samples. Normal bladder tissues (n = 7) were obtained from the archives of the Laboratory of Veterinary Pathology at the University of Tokyo, while iUC tissue samples (n = 10) were sourced from our laboratory’s tissue archives. The details were shown in the Supplementary S1 Table.

### Establishment of stably transduced cells

Canine iUC cells stably overexpressing the canine *HER2* (c*ERBB2*) gene were established through retroviral transduction. In brief, PLAT-gp cells were seeded in a 6-well plate, one day prior to transfection. A mixture of pMx-IP-cERBB2-#12 (made by Dr. Takuya Mizuno) and pCAGGS-VSVG was incubated with PEI Max (Polysciences, Warrington, PA, USA) and then added to the culture medium of PLAT-gp cells. The retroviral supernatant was collected and used to transduce iUC cells as previously described [14]. Transduced cells were selected using puromycin. As a control, we used cell lines expressing the empty vector.

We designed two target oligonucleotides (shRNA1, 5’- GCTCTTGGATGCGACGCAC −3’; shRNA2, 5’- CCGCTACTTTAACAAGAGG-3’) against the canine MCT4 gene and cloned them into the pSIREN-RetroQ vector (Clonetech) to generate MCT4 knockdown shRNA plasmids. As a control, we used the pSIREN-human DEPDC1B-kd2#1 plasmid (made by Dr. Takuya Mizuno), which does not bind to the canine genome sequence. MCT4-knockdown cell lines were generated using a retroviral transduction system as previously described [25].

### RNA sequencing (RNA-seq) analysis

We reanalyzed previously published fastq files (DRA005844) from 11 canine iUC tissues and 5 normal bladder tissues to evaluate the correlation between MCT4 expression and genes related to cellular metabolism. The fastq data were mapped to the canine genome assembly (CanFam3.1) using STAR v2.7.3a [26], and transcript abundance was quantified using RSEM v1.3.3 [27] with Ensembl gene transfer files (CanFam3.1.98, https://may2021.archive.ensembl.org/Canis_lupus_familiaris/). The raw expression count data were normalized using the multi-step trimmed mean of M values method, and then differentially expressed genes (DEGs) between the normal bladder and iUC were identified using estimateDE function in EdgeR (TCC v1.32.0) [28] and extracted based on a false discovery rate threshold of <0.01. The criteria for identifying upregulated and downregulated genes were based on absolute fold change (FC) between iUC and normal bladders (|FC| > 2), with a significance threshold set at *P*-value < 0.05. We performed downstream analysis to investigate the biological mechanisms and pathways associated with the identified genes. After filtering out low-expressed genes, the Ensembl gene IDs from the extracted genes were converted to HGNC symbols. GSEA was then conducted with clusterProfiler package (v4.6.2) in R (v4.2.1), referencing the Molecular Signatures Database (MSigDB v7.5.1).

### Protein extraction and western blotting

iUC cell lines were initially seeded in 6-well plates at a density of 3.0 × 10^5^ cells per well and then left to incubate overnight. After replacing the culture supernatant, cells were cultured for 24 hours. Subsequently, cells were harvested and lysed using NP40-based lysis buffer [consisting of 1% NP40, 10 mM Tris-HCl (pH 7.5), 150 mM NaCl, 1 mM EDTA, protease inhibitor cocktails (Nacalai Tesque, Kyoto, Japan), 1 mM Na_3_VO_4_, and 50 mM NaF] to extract whole proteins. After protein quantification, the samples underwent 10% acrylamide gel electrophoresis (SuperSep^TM^Ace, FujiFilm, Japan). The proteins were subsequently transferred onto a PVDF membrane (Merck, Darmstadt, Germany) and blocked with a blocking buffer consisting of Tris-buffered saline containing 0.05% Tween 20 and 5% nonfat milk for 1 hour at room temperature (RT). The membrane was incubated overnight at 4°C with primary antibodies, as shown in S2 Table. Following this, the membrane was washed and incubated with horseradish peroxidase (HRP)-conjugated anti-rabbit IgG secondary antibody (Jackson ImmunoResearch, West Baltimore Pike, West Grove, PA, USA) or HRP-conjugated anti-mouse IgG secondary antibody (BioLegend, San Diego, USA) for 1 h at RT. A mouse anti-β-actin monoclonal antibody was used as a loading control. Finally, specific proteins were visualized using an ECL reagent (PerkinElmer, Waltham, MA, USA) and subsequently detected with AMERSHAM Image Quant 800 (Global Life Sciences Technologies Japan, Tokyo, Japan). Western blot band intensities were quantified using ImageJ software, version 1.53 (National Institutes of Health, Bethesda, MD, USA).

### Measurement of lactic acid concentrations

iUC cells were cultured for 24 h, after which the culture supernatants were collected by centrifugation at 2,000 rpm for 10 minutes at 4°C. The lactic acid concentration was determined using the Lactic Acid-WST Assay Kit (DOJINDO). Absorbance at 450 nm was measured using an ARVO X4 fluorometer (PerkinElmer).

### Immunohistochemistry

Four-micrometer-thick sections of paraffin-embedded tissue were cut and mounted on MAS-coated glass slides (Matsunami Glass Kogyo, Osaka, Japan). After deparaffinization in xylene and rehydration in alcohol, antigen retrieval was performed by autoclaving in 0.01 M citrate buffer (pH 6.0) or Tris-EDTA (pH 9.0) for 20 min at 121°C. To block endogenous peroxidase activity, the sections were treated with 3% H_2_O_2_ in methanol for 30 min at RT, followed by rinsing with phosphate-buffered saline (PBS). Subsequently, sections were blocked with either 5% non-fat milk and bovine serum albumin in PBS or 5% non-fat milk in PBS for 30 minutes at RT to prevent non-specific binding. The sections were then incubated overnight at 4°C with primary antibodies, as detailed in S2 Table. After three 5-minute washes in PBS, the sections were incubated with Histofine Simple Stain MAX PO (Nichirei Corporation, Tokyo, Japan) as a secondary antibody for 30 minutes at RT. After another wash in PBS, brown staining was achieved using diaminobenzidine (Nacalai Tesque, Kyoto, Japan), followed by counterstaining with Mayer’s hematoxylin. MCT4 protein expression was quantified based on product intensity and positive staining rate. The intensity of diaminobenzidine and the positive rate were quantified by measuring the staining intensity and the brown-stained area, respectively, using ImageJ software, version 1.53 (National Institutes of Health).

ERBB2 immunoreactivity scoring was based on previous reports on canine iUC [29]. Samples were scored as follows: 0 (no reactivity), 1+ (weak and incomplete immunoreactivity in <10% of tumor cells), 2+ (strong but incomplete immunoreactivity in 10%≤ of tumor cells), and 3+ (strong and complete immunoreactivity in 10%≤ of tumor cells). According to a previous report [30], immunolabeled T cells were quantified in two distinct compartments: the intratumoral and peritumoral areas. The number of positively immunolabeled T cells was counted in these regions from 10 independent hotspots in HPFs, respectively. Additionally, the number of CD204-positive macrophages was measured using the same method as for the immunolabeled T cells. Next, we analyzed the correlation between MCT4 intensity and the number of immune cells using ImageJ software, version 1.53 (National Institutes of Health). All samples were reviewed and examined by a veterinary pathologist to confirm and validate the staining quality with all antibodies.

### ELISA

Peripheral blood mononuclear cells (PBMCs) were also isolated from healthy beagles maintained as blood donors at our veterinary teaching hospital, using density gradient centrifugation with Lymphoprep (Axis-Shield, Oslo, Norway). Isolated PBMCs were incubated in R10 medium for 1.5 h to allow monocyte adherence to culture plates, and only the non-adherent lymphocytes (PBLs) in the supernatant were collected. In the interferon (IFN)-γ production assay, PBLs were seeded at 2 × 10^5^ cells per well in U-bottom 96-well plates and stimulated with 2.5 μg/ml of ConA. L-lactic acid (MedChemExpress, New Jersey, USA) was added at 10 mM and incubated at 37°C in a CO_2_ incubator. After 72 hours of incubation, culture supernatants were collected and IFN-γ levels were measured using a canine IFN-γ DuoSet ELISA Development System (R&D Systems, Minneapolis, MN, USA). The cells from each sample were collected and stained with trypan blue.

### in vitro Treg differentiation

Naïve canine CD4^+^ T cells were isolated from PBLs using anti-canine CD4-FITC antibody (YKIX302.9, eBioscience, Inc., San Diego, CA) and sorted by the SH800 Cell Sorter (Sony Corporation, Tokyo, Japan). On day 0, CD4^+^ T cells were stimulated with plate-bound anti-canine CD3 monoclonal antibody (5 µg/mL, CA17.2A12, Bio-Rad Laboratories, Hercules, CA) and anti-canine CD28 monoclonal antibody (5 µg/mL, eBioscience) in the presence of recombinant human IL-2 (100 U/mL, Proleukin) and human TGF-β (5 ng/mL, Peprotech, Inc., Rocky Hill, NJ). After 3 days of incubation, cells were collected, counted, and reseeded at 5 × 10^5^ cells per well in 12-well plates, with IL-2 (300 U/mL) and TGF-β (5 ng/mL) being replenished. Subsequently, L-lactic acid (MedChemExpress) was added at a concentration of 10 mM. After 3 additional days, these cells were harvested and analyzed by flow cytometry (S1 Fig).

### Flow cytometry

Cell staining by flow cytometry was performed as described in a previous report [31]. To evaluate Treg differentiation, cells were incubated with anti-canine CD4-FITC antibody (YKIX302.9, eBioscience, Inc.) for 30 min on ice, washed with PBS, and then incubated with Fixable Viability Dye 660 (Invitrogen, Waltham, USA). The cells were incubated on ice for 30 min and washed twice with FACS buffer (PBS supplemented with 2% FCS and 0.1% NaN3). The cells were fixed and permeabilized using the Foxp3/Transcription Factor Staining Buffer Set (Invitrogen) according to the manufacturer’s instructions. Subsequently, the cells were stained with PE-conjugated anti-human Foxp3 antibody (clone FJK016s; eBioscience, Inc.) and analyzed using the CytoFLEX flow cytometer (Beckman Coulter, California, USA). Appropriate isotype controls were used for each sample. Flow cytometry data were analyzed using FlowJo software (Treestar, Inc., San Carlos, CA, USA). Experimental samples from experiments involving readouts on different days were analyzed on the same instrument with identical compensation settings to ensure comparability.

### Cell proliferation assay

For the cell proliferation assay, Sora and Love cells were seeded in triplicate in 96-well plates at a density of 2.0 × 10^4^ cells per well, while Nene cells were seeded at 1.0 × 10^4^ cells per well. The cells were treated with the indicated concentrations (6.25–200 μM) of NSAIDs, including aspirin, piroxicam, diclofenac (all from Selleck, Houston, USA), and robenacoxib (LGC, Middlesex, UK), for 48 hours. After treatment, Cell counting Kit-8 (DOJINDO) was added to each well, and cells were incubated for an additional 2 hours. After the 2-hour incubation period, absorbance at 450 nm was measured using an ARVO X4 fluorometer (PerkinElmer).

### Statistical analysis

Mean values and standard deviation (SD) were calculated from at least three independent biological experiments. To assess the significance of differences between samples, we performed a one-way factorial ANOVA followed by multiple comparisons using the Tukey-Kramer test. The data sets for IFN-γ quantification across the three treatment groups were compared using the Steel-Dwass test, while Treg differentiation between the two treatment groups was analyzed using the Matched Pairs test. Statistical significance was determined when *P*-values were less than 0.05. All comparison tests were conducted using JMP Pro software ver. 16.1.0 (SAS Institute Japan, Tokyo, Japan).

## Results

### Comprehensive gene expression analysis in iUC and healthy dogs

**Fig 1:**
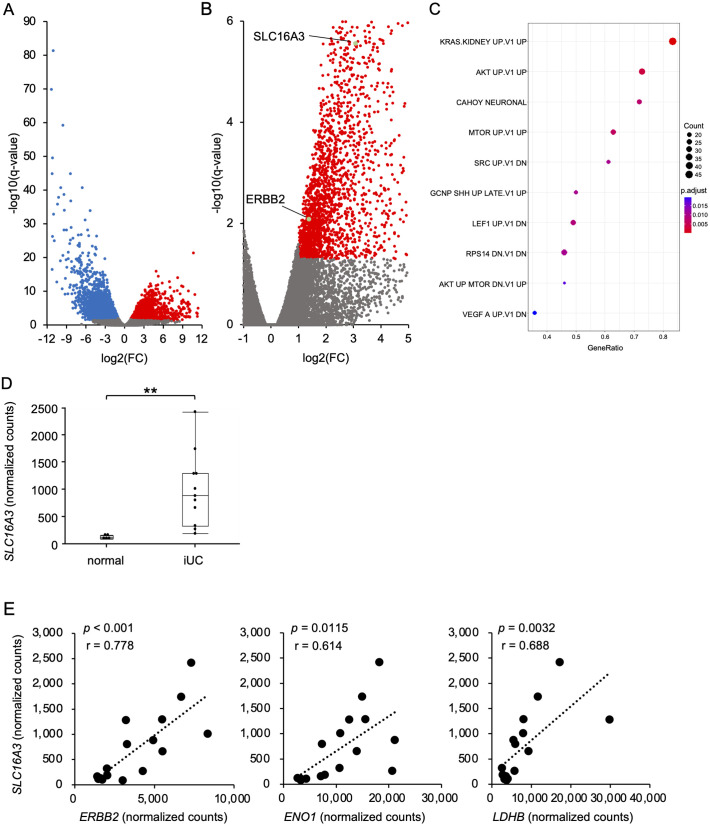
Comprehensive gene expression analysis in iUC and healthy dogs. (A, B) Volcano plot of differentially expressed genes in iUC versus normal bladder. (C) GSEA data indicated the upregulated signaling pathways in iUC. (D) Box plots depicting normalized counts of *SLC16A3* (MCT4) between iUC and normal bladder are shown. Wilcoxon/Kruskal-Wallis test, **p < 0.01. (E) Correlations between the *SLC16A3* gene and genes involved in cellular metabolism (*ERBB2*, *ENO1*, and *LDHB*) are shown.

To investigate why MCT4 is highly expressed in iUC [14], we reanalyzed previously published RNA-seq data from 11 canine iUC cases and five normal bladder tissues [32]. To assess differential gene expression between iUC and normal bladder tissues, we generated volcano plots (Fig 1A and 1B). We identified 7,979 significantly upregulated and 8,020 downregulated genes in iUCs compared to normal bladders. Among these, we extracted the glycolysis-related genes in iUCs (Table 1). Gene set enrichment analysis (GSEA) analysis revealed upregulation of MAPK and PI3K pathways in iUC (Fig 1C). *SLC16A3* normalized counts were significantly higher in iUC compared to normal bladder tissue (Fig 1D). When gene expression related to these pathways was compared with *SLC16A3* gene expression, normalized counts of *SLC16A3* were positively correlated with those of *ERBB2* (Fig 1E). The comparison of cell metabolism-related genes and *SLC16A3* revealed a positive correlation between the gene expression of *SLC16A3* and those of *ENO1* (r = 0.614, *p* = 0.0115) and *LDHB* (r = 0.688, *p* = 0.0032), both of which are associated with glycolysis (Fig 1E).

**Table 1:** The list of upregulated and downregulated glycolysis-related genes in canine iUCs.

Genes	Mean of iUC*	Mean of control**	Fold change (log2)	P value	q value
HIF3A	2.950	154.001	−5.706	<0.00001	<0.00001
SLC16A3	988.321	117.875	3.068	<0.00001	<0.00001
ERBB3	8006.881	1580.335	2.341	<0.00001	<0.00001
LPAR4	1.377	25.346	−4.202	<0.00001	2.51.E-05
AKT3	269.460	1457.356	−2.435	<0.00001	4.38.E-05
ENO1	13914.737	4125.896	1.754	1.09.E-05	1.95.E-04
LPAR2	77.715	10.620	2.871	1.10.E-05	1.97.E-04
PFKM	440.200	1448.399	−1.718	4.47.E-05	6.51.E-04
LPAR1	169.870	474.060	−1.481	6.89.E-05	9.38.E-04
ENO3	63.911	221.850	−1.795	7.07.E-05	9.58.E-04
ENO2	118.297	507.522	−2.101	1.56.E-04	1.87.E-03
PFKL	6918.282	1432.678	2.272	5.48.E-04	5.39.E-03
ERBB2	4829.976	1851.981	1.383	9.04.E-04	8.19.E-03
PFKFB2	367.338	140.922	1.382	2.44.E-03	1.85.E-02
LDHB	9617.542	3503.363	1.457	4.23.E-03	2.87.E-02
MYCBPAP	20.672	65.126	−1.656	6.23.E-03	3.90.E-02
MYCL	612.443	235.464	1.379	8.43.E-03	4.92.E-02
MYCT1	105.693	254.269	−1.266	1.38.E-02	7.25.E-02
PFKFB3	1351.940	603.700	1.163	2.00.E-02	9.64.E-02
PFKFB1	0.308	4.714	−3.937	2.12.E-02	1.01.E-01
NADSYN1	582.297	325.568	0.839	2.38.E-02	1.10.E-01
AKT2	767.712	1199.944	−0.644	3.61.E-02	1.50.E-01
HK1	5227.438	2949.797	0.825	4.60.E-02	1.81.E-01
HIF1A	3445.917	5610.295	−0.703	4.90.E-02	1.90.E-01
TP53	1955.111	1170.498	0.740	5.22.E-02	1.98.E-01
HK3	34.773	10.702	1.700	5.68.E-02	2.12.E-01
SLC16A1	322.542	759.163	−1.235	6.12.E-02	2.23.E-01
PTEN	808.594	1131.492	−0.485	1.60.E-01	4.47.E-01
CA9	40.195	81.274	−1.016	2.14.E-01	5.54.E-01
ERBB4	4.407	0.447	3.301	2.34.E-01	5.87.E-01
LPAR3	25.500	39.634	−0.636	3.57.E-01	7.75.E-01
ENO4	0.263	0.863	−1.714	3.60.E-01	7.80.E-01
GAPDH	20246.910	15605.507	0.376	3.61.E-01	7.81.E-01
PFKP	1993.360	2545.325	−0.353	3.86.E-01	8.18.E-01
LPAR6	121.416	84.191	0.528	4.49.E-01	9.09.E-01
LDHD	138.460	94.715	0.548	4.81.E-01	9.53.E-01
MYC	1231.773	1458.091	−0.243	5.01.E-01	9.79.E-01
AKT1	3164.853	2697.503	0.231	5.56.E-01	1.00.E + 00
HK2	262.142	192.043	0.449	5.83.E-01	1.00.E + 00
GAPDHS	3.520	2.727	0.368	7.05.E-01	1.00.E + 00
NADK	1174.774	1112.382	0.079	8.25.E-01	1.00.E + 00
NADK2	324.036	334.093	−0.044	8.72.E-01	1.00.E + 00
EGFR	2188.385	2101.106	0.059	9.00.E-01	1.00.E + 00

*Mean normalized counts of iUC.

**Mean normalized counts of normal bladder tissues.

### Protein expressions of ERBB2 signaling pathway in iUC cells

**Fig 2:**
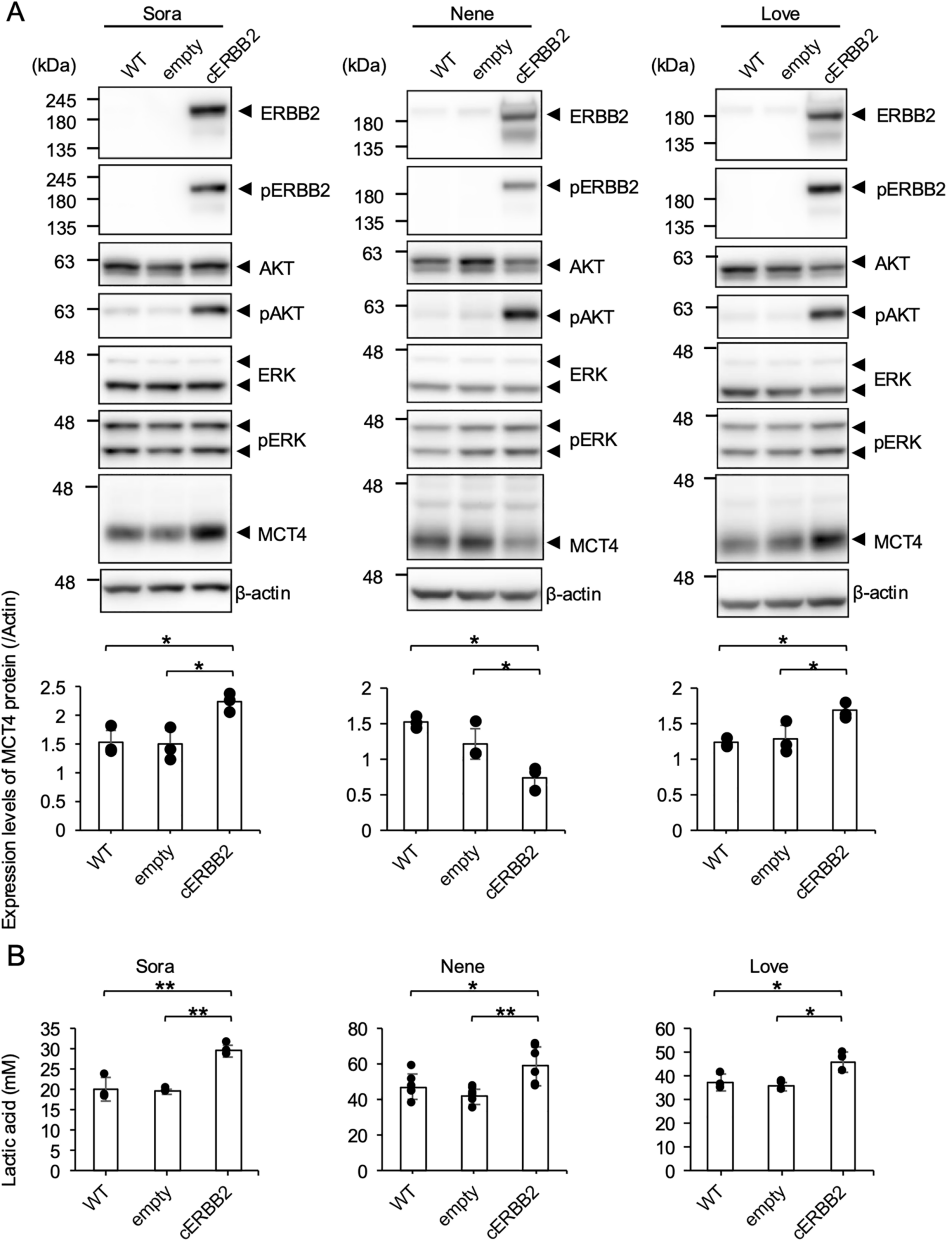
ERBB2 activates AKT signaling and glycolysis in iUC cells. (A) Protein expression levels of ERBB2, AKT, ERK, and MCT4 in ERBB2-overexpressing iUC cell lines are shown. Cropped images of western blotting results are presented. The relative expression level of MCT4 was normalized to that of β-actin based on the results of three independent experiments. Mean ± SD is shown. Tukey-Kramer test, *p < 0.05. (B) Extracellular lactic acid levels in Sora/cERBB2 and Love/cERBB2 was evaluated in three independent experiments; Nene/cERBB2 was evaluated in six independent experiments. Mean ± SD is shown. Turkey–Kramer test, *p < 0.05. **p < 0.01.

To investigate the correlation between ERBB2 activation and MCT4 expression, we analyzed protein levels by western blotting in three iUC cell lines (Sora, Nene, and Love), which were previously confirmed to harbor wild-type *ERBB2* gene [24]. Overexpression of the *ERBB2* gene induces ERBB2 protein dimerization at the cell membrane, leading to downstream signaling pathway activation [33]. Subsequently, we established iUC cell lines stably overexpressing canine ERBB2 (cERBB2) protein. AKT phosphorylation was upregulated in all iUC/cERBB2 cell lines, while ERK phosphorylation remained unchanged. ERBB2 signaling pathway activation increased MCT4 protein expression levels in Sora/cERBB2 and Love/cERBB2 cells while decreasing MCT4 expression in Nene/cERBB2 cells (Fig 2A). To investigate whether ERBB2 activation affects glycolysis, we measured lactic acid concentrations in the culture supernatant of each cell line. All iUC, Sora/cERBB2, Nene/cERBB2, and Love/cERBB2 cells exhibited significantly higher extracellular lactic acid levels compared to wild-type and empty vector controls (Fig 2B). ERBB2 overexpression led to AKT upregulation and enhanced glycolytic activity in iUC cells, corroborating the RNA-seq findings.

### MCT4 knockdown impairs lactic acid secretion

**Fig 3:**
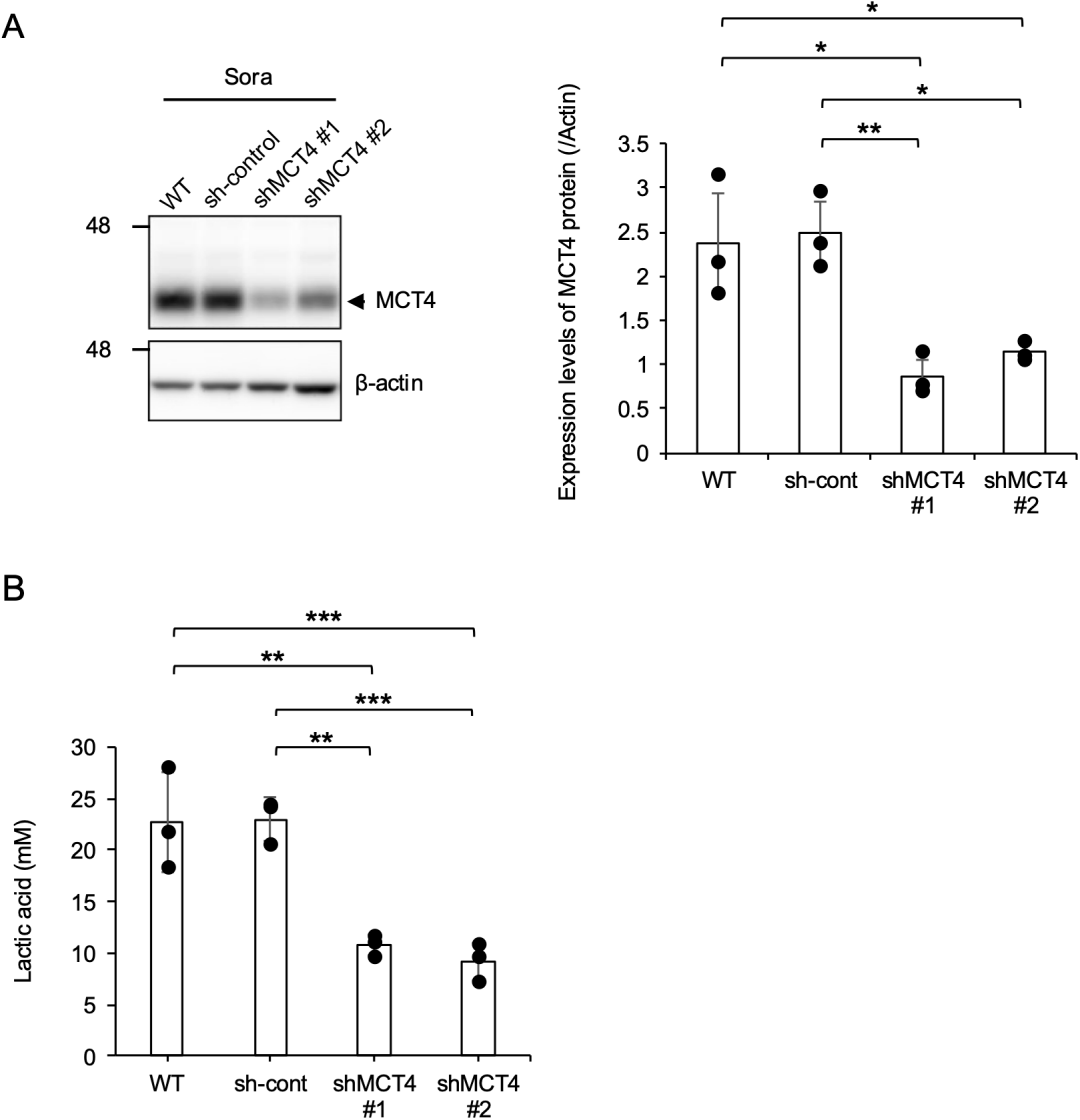
MCT4 knockdown impairs lactic acid secretion. (A) MCT4 protein expression levels in Sora/shMCT4#1 and #2 are shown. Cropped images of western blotting results are presented. The relative expression level of MCT4 was normalized to that of β-actin based on the results of three independent experiments. Mean ± SD is shown. Turkey-Kramer test, *p < 0.05. **p < 0.01. (B) Extracellular lactic acid levels of Sora/shMCT4#1 and #2 were evaluated in three independent experiments. Mean ± SD is shown. Turkey–Kramer test, **p < 0.01. ***p < 0.001.

To validate MCT4’s role in extracellular lactic acid levels, two MCT4 gene knockdown cell lines (shMCT4#1 and shMCT4#2) were established from Sora and Nene cells. MCT4 protein expression was downregulated in both Sora/shMCT4#1 and #2 cells compared to wild-type (WT) and sh-control cells. MCT4 expression in sh-control cells did not differ from that of the parental cells (Fig 3A). MCT4 protein downregulation in Sora/shMCT4#1 and #2 cell lines resulted in significantly decreased extracellular lactic acid levels compared to WT or sh-control cells (Fig 3B). In contrast to the findings in Sora cells, MCT4 knockdown did not suppress extracellular lactic acid levels in Nene cells (S2A–B Fig). Similar to Fig 2A, this indicates that extracellular lactic acid levels in Nene cells are independent of MCT4 protein expression.

### MCT4 protein expression positively correlates with the number of Foxp3 + Treg cells in iUC tissues

**Fig 4:**
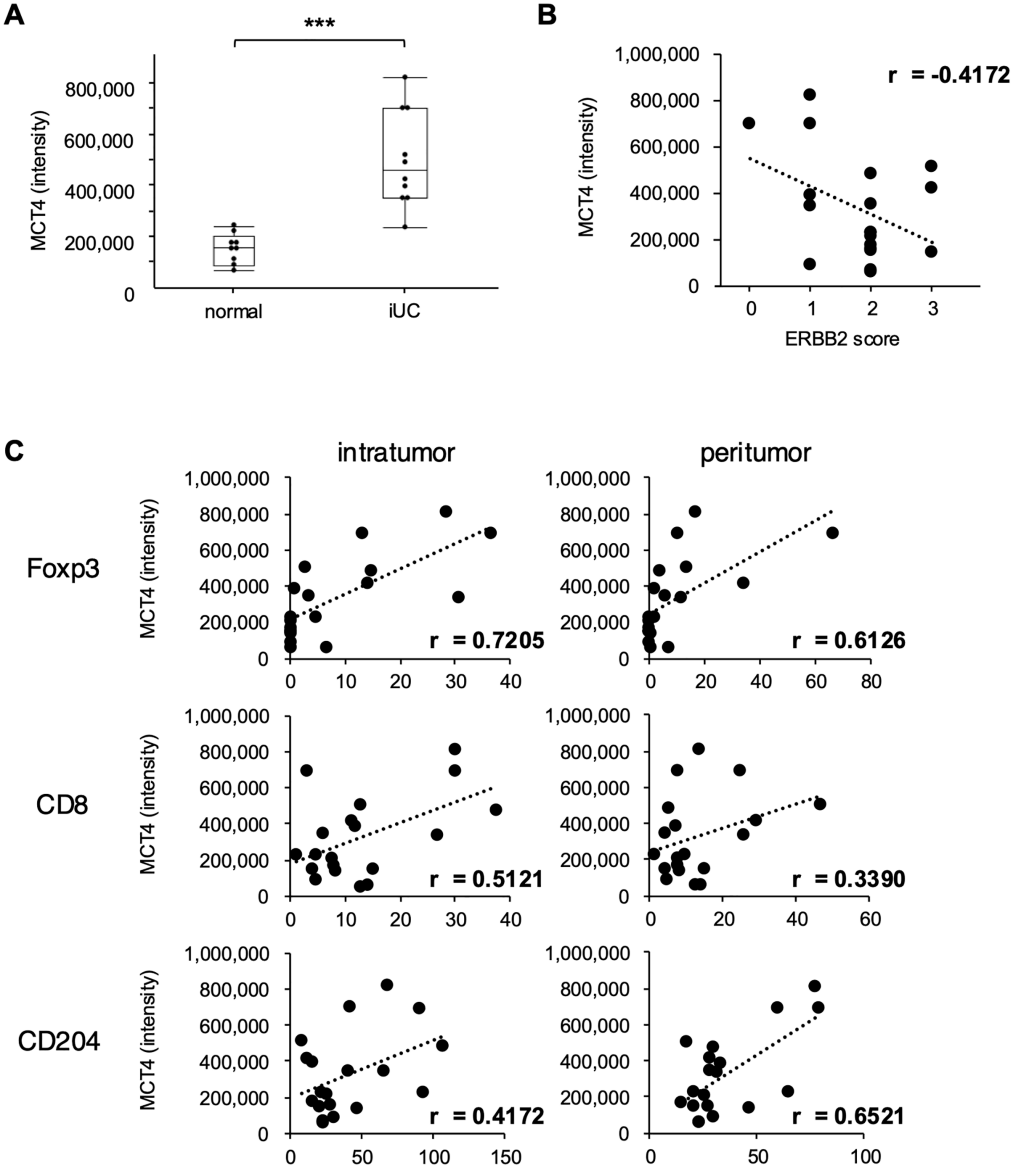
The expression levels of MCT4 protein were positively correlated with the number of Foxp3^+^ Treg cells in iUC tissues. (A) Box plots for expression levels of MCT4 in iUC and normal bladder tissues are shown. Wilcoxon/Kruskal-Wallis test, ***p < 0.001. (B) Correlations between MCT4 expression intensity and ERBB2 immunoreactive score are shown. (C) Correlations between MCT4 intensity and immune cell infiltration (Foxp3, CD8, CD204) are shown. The respective results for intratumoral and peritumoral regions are shown.

To compare MCT4 expression levels in iUC and normal bladder tissues, we performed immunohistochemistry and quantitative image analysis on 10 iUC and 9 normal bladder tissues. Representative images of the immunohistochemical staining are provided in S3 Fig. Quantitative image analysis revealed that the mean signal density in MCT4-positive areas (expression intensity) was significantly higher in iUC tissue (494.232 ± 45.303) compared to normal bladder tissue (143.253 ± 47.753) (Fig 4A). ERBB2 immunoreactivity scores for each sample are detailed in Supplementary S1 Table. In the iUCs, 2 samples scored 3, 3 samples scored 2, 4 samples scored 1, and 1 sample scored 1. In normal bladder tissue, 1 sample scored 3, 1 sample scored 1, and the remaining samples scored 2. There was no positive correlation between MCT4 and ERBB2 expression levels (*p* = 0.1960, r = −0.4172) (Fig 4B).

Subsequently, to evaluate the relationship between immune cell infiltration and MCT4 expression, we performed immunohistochemical staining of iUC tissues using T-cell markers (Foxp3 and CD8) and an M2 macrophage marker (CD204). Representative IHC images are shown in Supplementary S3 Fig. The number of intratumoral and peritumoral Foxp3-positive T-cells was 8.13 ± 11.69/high-power fields (HPF) and 9.03 ± 16.43/HPF, respectively. The number of intratumoral and peritumoral CD8-positive T-cells was 12.99 ± 10.41/HPF and 13.03 ± 11.31/HPF, respectively. The number of CD204-positive intratumoral and peritumoral macrophages was 40.33 ± 29.76/HPF and 35.64 ± 19.88/HPF, respectively. MCT4 expression levels positively correlated with both intratumoral and peritumoral Foxp3-positive T-cell counts (r = 0.7205 and r = 0.6126, respectively). Furthermore, there was a positive correlation between MCT4 expression levels and CD204-positive peritumoral macrophages (r = 0.6521), but not between CD8-positive T-cells and CD204-positive intratumoral macrophages (Fig 4C). These findings indicate that, within the TME, Foxp3-positive Treg cell infiltration showed the strongest correlation with MCT4 expression among all immune cells.

### Lactic acid promotes Treg differentiation in dogs

**Fig 5:**
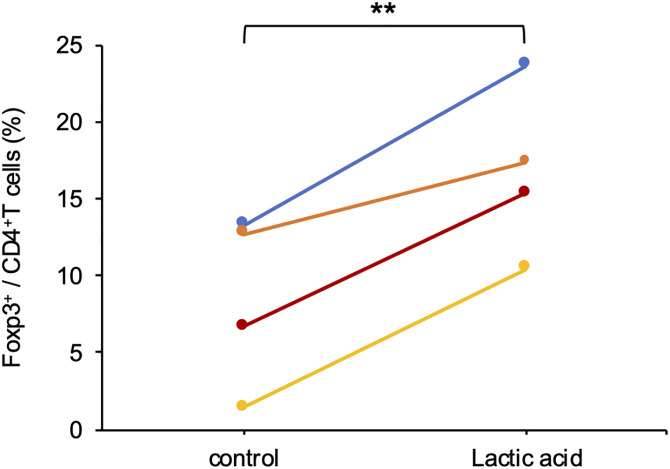
Lactic acid increases Treg differentiation in dogs. The ratio of Foxp3-positive cells to CD4-positive T cells is shown. Each color bar represents a different individual. Matched Pairs test, **p < 0.01.

To investigate the effect of lactic acid on Treg cells, we performed an *in vitro* Treg differentiation assay. Naïve CD4^+^ T cells were isolated from PBLs and stimulated with TGF-β, IL-2, and anti-CD3/CD28 antibodies in the presence or absence of lactic acid (10 mM). After 3 days of induction, Foxp3^+^/CD4^+^ T cells was significantly upregulated in the lactic acid group compared to controls (Fig 5). Therefore, lactic acid increased the absolute number of CD4^+^Foxp3^+^ Treg cells in canine T cells.

### Lactic acid suppressed IFN-γ production in mitogen-stimulated lymphocytes.

**Fig 6:**
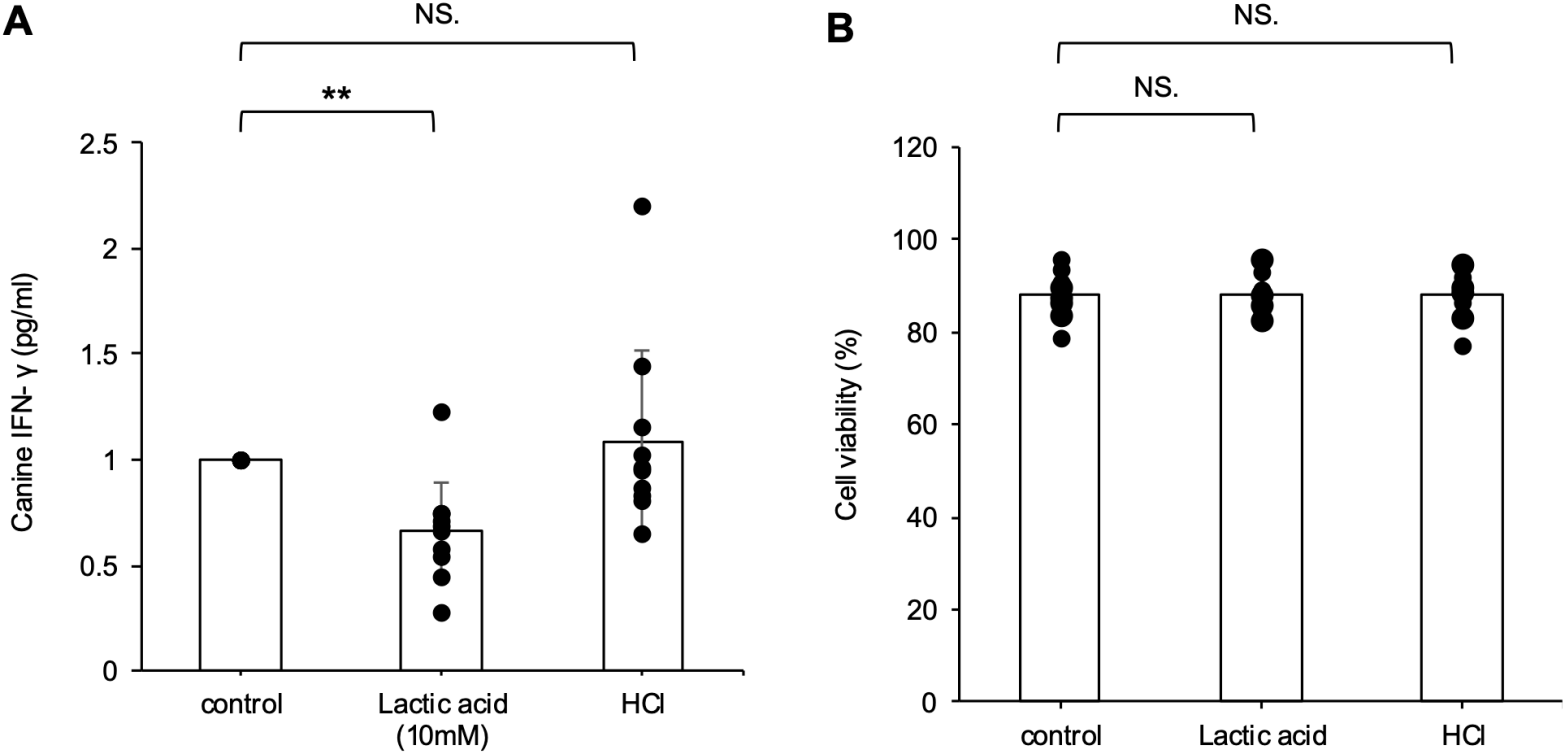
Lactic acid suppressed IFN-γ production in mitogen-stimulated lymphocytes. (A) the amount of canine IFN-γ in the supernatants was measured in duplicate. (B) The cells were collected and trypan blue staining was performed to assess cell viability. Steel-Dwass test, **p < 0.01. NS, not significant.

To examine the effect of lactic acid on canine lymphocyte function, we measured the concentration of IFN-γ released from stimulated PBLs in the culture supernatant. PBLs collected from 10 healthy dogs were stimulated with ConA and cultured with lactic acid (10 mM) for 3 days. HCl was used as a control to reduce the pH of the medium to a similar extent as lactic acid. IFN-γ production was significantly reduced in the presence of lactic acid but not HCl (Fig 6A). Cell viability remained unaffected by either lactic acid or HCl (Fig 6B).

### Effect of NSAIDs on cell proliferation and lactic acid metabolism in iUC cell lines

**Fig 7:**
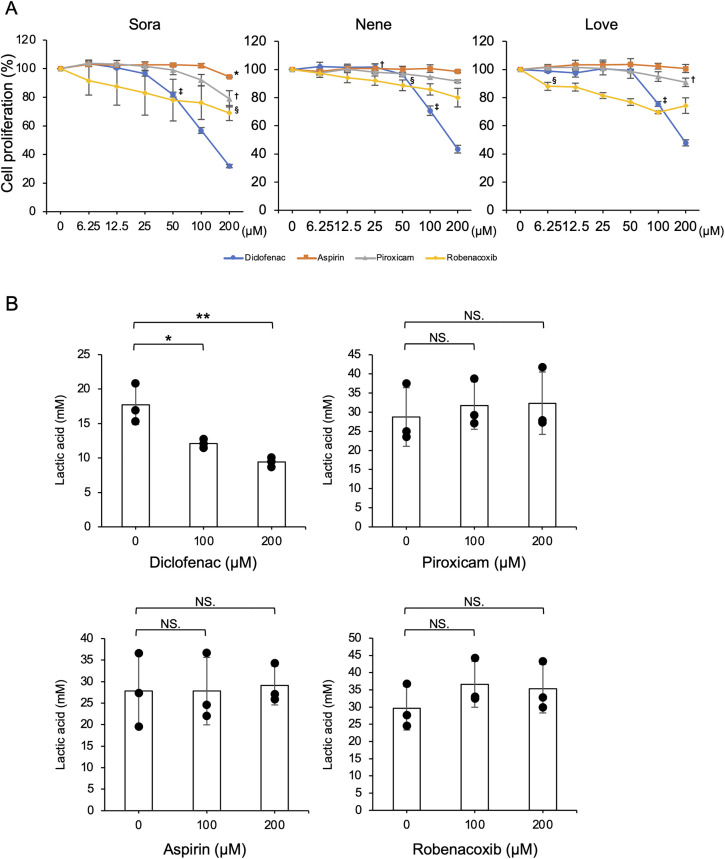
Effect of NSAIDs on cell proliferation and extracellular lactic acid levels in iUC cell lines. (A) Cell proliferation assay results using NSAIDs (aspirin, piroxicam, diclofenac, and robenacoxib). The iUC cell lines were seeded in triplicate in the presence of NSAIDs at the indicated concentrations for 48 h, and cell proliferation rates were determined using the Cell Counting Kit-8 assay. The proliferation rate of NSAID-treated cells at each concentration is expressed relative to DMSO-treated control cells, set at 100%. The results were evaluated in three independent experiments. Mean ± SD is shown. Statistically significant differences compared to the DMSO-treated control group were first observed at a certain concentration, and all higher concentrations were also considered significantly different. Dunnett test, *p* < 0.05. These concentrations are indicated in the figure: *aspirin, ^†^piroxicam, ^‡^diclofenac, ^§^robenacoxib. (B) Results of extracellular lactic acid levels using NSAIDs. The iUC cell lines were seeded in the presence of NSAIDs at concentrations of 100 or 200 μM for 24 h, and extracellular lactic acid levels was measured using a lactic acid assay kit. The results were evaluated in three independent experiments. Mean ± SD is shown. Tukey-Kramer test, *p < 0.05, **p < 0.01. NS, not significant.

To evaluate the cytotoxic effects of NSAIDs (aspirin, piroxicam, diclofenac, and robenacoxib), each iUC cell line was cultured with varying NSAID concentrations (6.25−200 µM) for 48 hours. Aspirin (non-selective COX inhibitor), piroxicam (with reported antitumor effects in canine iUC [3]), diclofenac (reported MCT4 inhibitor in humans [34]), and robenacoxib (selective COX-2 inhibitor structurally related to diclofenac and used in veterinary practice) were selected based on clinical relevance and prior evidence. Diclofenac and robenacoxib inhibited cell proliferation in a dose-dependent manner, whereas aspirin and piroxicam did not (Fig 7A). Subsequently, only diclofenac significantly reduced extracellular lactic acid levels in Sora (Fig 7B) and Nene (S2C Fig).

## Discussion

RNA-seq data reanalysis revealed that canine iUCs had high *SLC16A3* expression and its correlation with *ERBB2* expression and glycolytic markers. Therefore, we investigated the molecular mechanism of glycolysis using ERBB2-overexpressing iUC cell lines. ERBB2 overexpression increased AKT phosphorylation without ERK phosphorylation, resulting in enhanced extracellular lactic acid levels in canine iUC cells. This indicated that ERBB2 downstream pathway in canine iUC relied on the PI3K/AKT pathway, although ERBB2 could regulate multiple downstream pathways, such as MAPK and SRC, in human cancers [35]. In Sora and Love, activation of the ERBB2-AKT-glycolysis axis led to the upregulation of MCT4. Conversely, in Nene, although ERBB2 overexpression resulted in increased extracellular lactic acid levels, MCT4 expression was reduced. Other MCT family members, such as MCT1, may transport intracellular lactic acid into the culture supernatant [36]. According to this hypothesis, Nene cells constitutively expressed MCT1, and extracellular lactic acid levels remained unchanged following MCT4 knockdown. Notably, Nene cells exhibited lower expression of MCT4 and higher expression of MCT1 compared to Sora and Love cells (S4A Fig). Furthermore, ERBB2 overexpression in Nene cells led to a further reduction in MCT4 expression, accompanied by an increase in MCT1 expression (S4B Fig). These results suggest that the contribution of different monocarboxylate transporters to lactic acid export may vary among cell lines, and that MCT1 may play a compensatory or alternative role in lactic acid handling in Nene cells.

Immunohistochemistry revealed no positive correlation between MCT4 expression levels and ERBB2 expression scores in iUC and normal bladder tissues. However, we did not evaluate *ERBB2* amplification and mutation in those samples. A previous report shows that *ERBB2* copy number aberration was detected in iUC; however, no significant association was found between *ERBB2* copy number aberration and protein expression [21]. Therefore, we suggest that the lack of correlation between MCT4 and ERBB2 expression in our findings should be evaluated in conjunction with *ERBB2* sequencing. Another factor to consider is that ERBB2 expression scores in normal bladder tissues used in our study were higher than previously reported [29], which may explain the lack of significant difference between these and iUC tissues. Tsuboi et al., [29] used polyclonal anti-human ERBB2 antibody (DAKO, A0485), whereas we used monoclonal anti-human ERBB2 antibody (Clone CB11) for immunohistochemistry. Human studies have shown that the monoclonal antibody (CB11) have higher specificity for ERBB2 protein compared to the polyclonal antibody (A0458) [37]. RNA-seq data also revealed that *ERBB2* expression was approximately 2.6-fold higher in iUC tissues compared to normal bladder tissues. This difference is relatively small compared to MCT4, suggesting that ERBB2 expression alone may not be crucial in distinguishing between normal and tumor tissues. Furthermore, the staining method may have influenced the ERBB2 immunoreactivity scores, potentially explaining why our findings diverged from a previous report [29]. Nevertheless, further investigation of ERBB2 genomic status in formalin-fixed, paraffin-embedded (FFPE)-iUC tissues is needed to validate our *in vitro* model and expand the sample size.

A high concentration of lactic acid in the TME enhances Treg function and suppresses effector T cell activity, ultimately leading to the inhibition of antitumor immunity [38]. In canine iUC tissues, MCT4 expression levels had a strong positive correlation with Treg cell counts, but not with other immune cells. This suggests that lactic acid secreted by tumor cells may affect immune cells infiltrating iUC tissues through Treg cell activity; however, its specific effects on differentiation, proliferation, and function of these cells remain unclear. Therefore, we evaluated the effects of lactic acid on Tregs and effector T cells using *in vitro* lymphocyte culture models.

We found that exogenous lactic acid promotes Treg differentiation and suppresses IFN-γ production by effector T cells in dogs, which is consistent with previous reports [6,7]. Although exogenous lactic acid promoted canine Treg differentiation, the induction efficiency was lower than that reported for human and mouse Tregs, with approximately 20% of Foxp3 ⁺ cells observed in canine lymphocytes compared to 40–60% in previous human and mouse studies [7,39]. In the present study, human-derived products were used, including human IL-2 and human TGF-β, which may have reduced Treg differentiation efficiency. Despite previous reports indicating that acidic pH severely inhibits effector T cell function [40], HCl alone did not suppress IFN-γ production, unlike lactic acid. Thus, the suppressive effects of lactic acid on effector T cells are not solely attributed to a decrease in pH but may involve specific metabolic pathways or signaling pathways activated by lactic acid uptake into T cells. Research on canine Tregs remains scarce, and their function remains unclear. Therefore, this report is the first study on Treg differentiation in dogs.

MCT4 inhibition reduces lactic acid secretion and has emerged as a promising therapeutic strategy in human cancers [41,42]. Similar to human findings, MCT4 knockdown in the canine iUC cell line Sora, but not in Nene, suppressed extracellular lactic acid levels in the culture supernatant. Therefore, lactic acid excretion may vary among cell lines due to differences in the primary transporters being used. Furthermore, diclofenac showed mild growth-inhibitory effects and significantly reduced extracellular lactic acid levels in canine iUC cell lines (Fig 7 and S2C Fig). In humans, diclofenac acts as a potent inhibitor of MCT1 and MCT4, unlike other NSAIDs, thereby reducing lactic acid secretion. Diclofenac reduces lactic acid independently of changes in glycolysis-related proteins and MCT expression levels [34]. Furthermore, studies in human and mouse models have demonstrated that suppressing lactic acid secretion through diclofenac effectively counteracts the immunosuppressive effects on antitumor immunity in the TME. This encompasses maintaining effector T cell function, promoting IL-12 production, reducing IL-10 secretion by dendritic cells, and decreasing tumor-infiltrating Tregs [34,43]. In addition, a recent study has shown that diclofenac may also inhibit lactate dehydrogenase A (LDHA), a key enzyme in the final step of glycolysis, by binding allosterically near its substrate-binding site [44]. This dual mechanism, blocking both lactic acid production and export, might explain the significant reduction in lactic acid observed in our study. While we did not directly assess LDHA activity in canine iUCs, future studies should explore whether diclofenac affects glycolytic enzyme activity in this context. Therefore, in dogs, diclofenac may serve as an MCT4 inhibitor, and its suppression of extracellular lactic acid levels in the TME could potentially restore antitumor immunity. These findings suggest that diclofenac may represent a novel therapeutic alternative to piroxicam for the treatment of canine iUC.

## Conclusion

Our findings demonstrate that ERBB2-AKT pathway activation increased the lactic acid efflux alongside MCT4 expression in iUC. Furthermore, exogenous lactic acid increases Treg differentiation and inhibits IFN-γ production in canine T cells. Therefore, diclofenac, an MCT4 inhibitor, may represent a novel therapeutic approach for iUC. Given the histopathological and molecular similarities between canine iUC and human muscle-invasive bladder cancer, our findings may also have translational relevance. Spontaneous canine iUC provides a useful comparative model for investigating tumor metabolism and immune regulation, potentially contributing to the development of novel therapeutic strategies in human oncology. However, this study has several limitations. The limited sample size precludes the investigation of the association between MCT4 expression in canine FFPE tumor tissues and prognosis. In addition, while increased lactic acid levels were observed in the culture supernatant, this does not necessarily indicate enhanced production, as they may reflect increased efflux. Glucose consumption was not measured, which limits the interpretation of glycolytic activity. Since the Treg culture system has not been fully established in dogs, it is essential to increase Treg differentiation efficiency and conduct a thorough investigation of Treg function. However, this study suggests that targeting tumor metabolism may represent a promising therapeutic approach for canine tumors.

## Supporting information

S1 Fig Representative results of the flow cytometry are displayed. Naive canine CD4 + T cells were sort-purified from PBLs by Cell Sorter SH800 (Sony Corporation, Tokyo, Japan). Tregs were defined as CD4-positive and Foxp3-positive cells, and the proportion of Foxp3-positive cells was calculated in comparison to the isotype control. The proportion of Foxp3-positive cells within the CD4-positive cell population was defined as the Treg ratio.
(TIFF)

S2 Fig The inhibition of MCT4 effect on Nene of canine iUC cell line. (A) Protein expressions of MCT4 in Nene/shMCT4#1 and #2 are shown. Cropped images of western blotting results are presented. The relative expression level of MCT4 was normalized to that of β-actin based on the results of three independent experiments. Mean ± SD is shown. Turkey-Kramer test, *p < 0.05. **p < 0.01. ***p < 0.001. N.S., not significant. (B) Extracellular lactic acid levels of Nene/shMCT4#1 and #2 were evaluated in three independent experiments. Mean ± SD is shown. Turkey–Kramer test, NS., not significant. (C) Results of extracellular lactic acid levels using NSAIDs. The iUC cell lines (Nene) were seeded in the presence of Diclofenac at 100 or 200 µM for 24 h, and the extracellular lactic acid levels was measured by lactic acid assay kit. The results were evaluated in three independent experiments. Mean ± SD is shown. Tukey-Kramer test, *p < 0.05, **p < 0.01.
(TIFF)

S3 Fig Representative results of the IHC are displayed. The results of IHC in iUC tissues. Scale bar: 50 µm.
(TIFF)

S4 Fig MCT1 expression in Nene of canine iUC cell line. (A) Protein expressions of MCT4 and MCT1 in iUC cell lines are shown. Cropped images of western blotting results are presented. (B) Protein expressions of MCT4 in Nene/cERBB2 is shown. Cropped images of western blotting results are presented. The relative expression level of MCT1 was normalized to that of β-actin based on the results of three independent experiments. Mean ± SD is shown. Turkey-Kramer test, *p < 0.05. **p < 0.01.
(TIFF)

S1 Table Baseline subject characteristics in iUC and normal bladder tissues. (XLSX)

S2 Table Antibodies used in this study. (XLSX)
