# Supplementary figures and images for: Lactic acid regulates antitumor immunity in canine invasive urothelial carcinoma
Source: PLoS One. 2025 Sep 18;20(9):e0332825. doi: 10.1371/journal.pone.0332825 (PMC12445541; doi:10.1371/journal.pone.0332825)

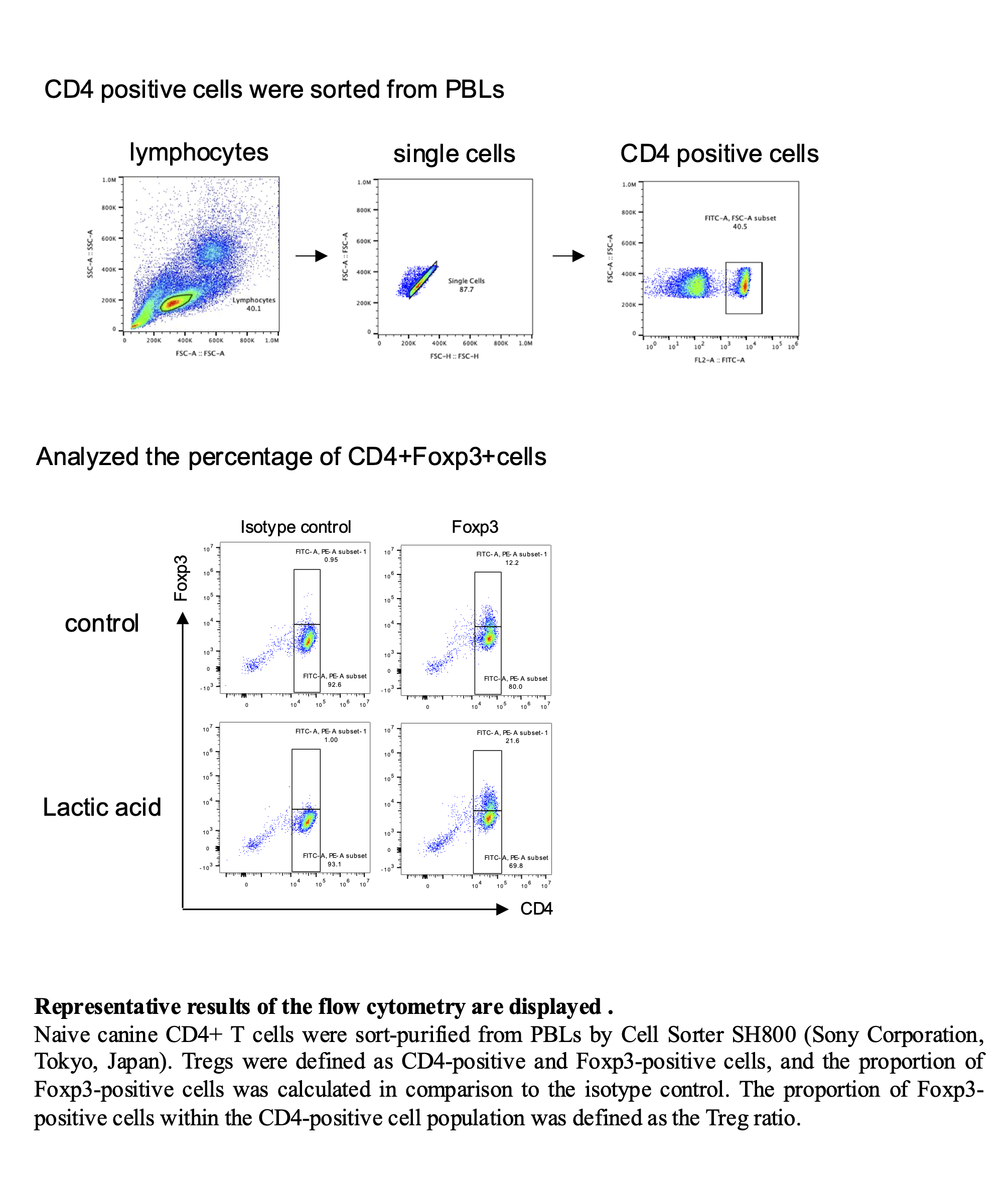

Supplement: S1 Fig — Naive canine CD4 + T cells were sort-purified from PBLs by Cell Sorter SH800 (Sony Corporation, Tokyo, Japan). Tregs were defined as CD4-positive and Foxp3-positive cells, and the proportion of Foxp3-positive cells was calculated in comparison to the isotype control. The proportion of Foxp3-positive cells within the CD4-positive cell population was defined as the Treg ratio. (TIFF) [file pone.0332825.s001.tiff]

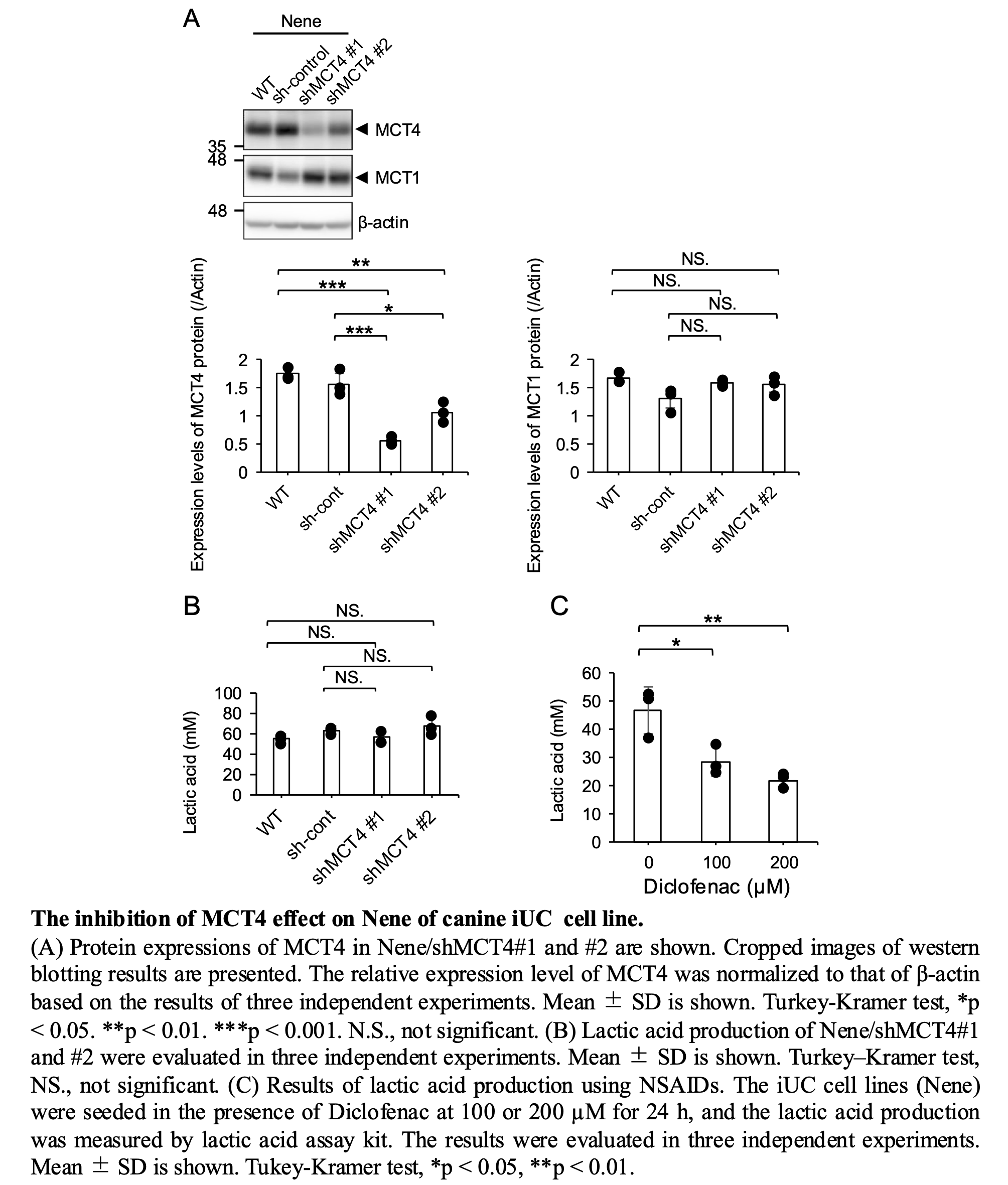

Supplement: S2 Fig — (A) Protein expressions of MCT4 in Nene/shMCT4#1 and #2 are shown. Cropped images of western blotting results are presented. The relative expression level of MCT4 was normalized to that of β-actin based on the results of three independent experiments. Mean ± SD is shown. Turkey-Kramer test, *p < 0.05. **p < 0.01. ***p < 0.001. N.S., not significant. (B) Extracellular lactic acid levels of Nene/shMCT4#1 and #2 were evaluated in three independent experiments. Mean ± SD is shown. Turkey–Kramer test, NS., not significant. (C) Results of extracellular lactic acid levels using NSAIDs. The iUC cell lines (Nene) were seeded in the presence of Diclofenac at 100 or 200 µM for 24 h, and the extracellular lactic acid levels was measured by lactic acid assay kit. The results were evaluated in three independent experiments. Mean ± SD is shown. Tukey-Kramer test, *p < 0.05, **p < 0.01. (TIFF) [file pone.0332825.s002.tiff]

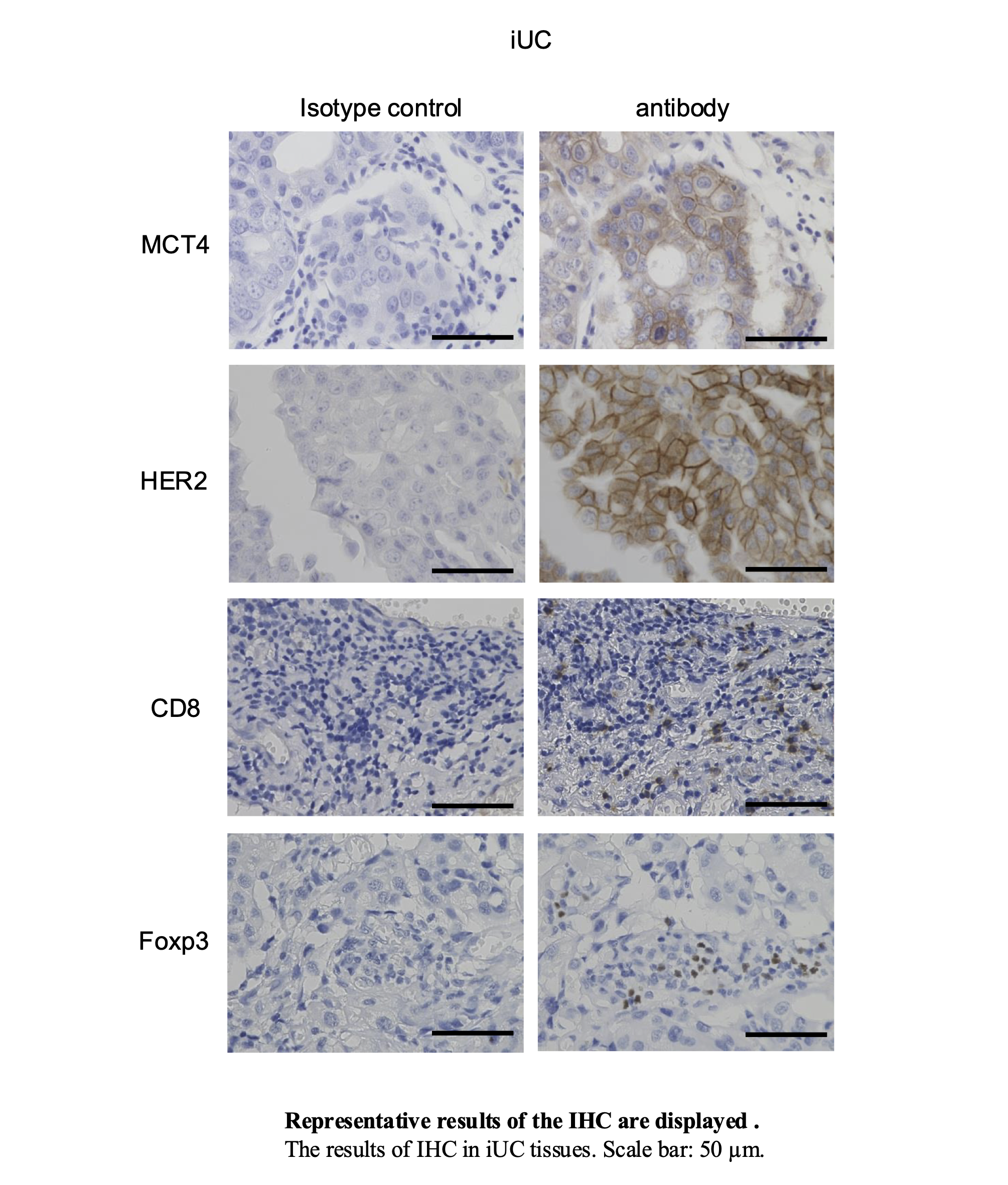

Supplement: S3 Fig — The results of IHC in iUC tissues. Scale bar: 50 µm. (TIFF) [file pone.0332825.s003.tiff]

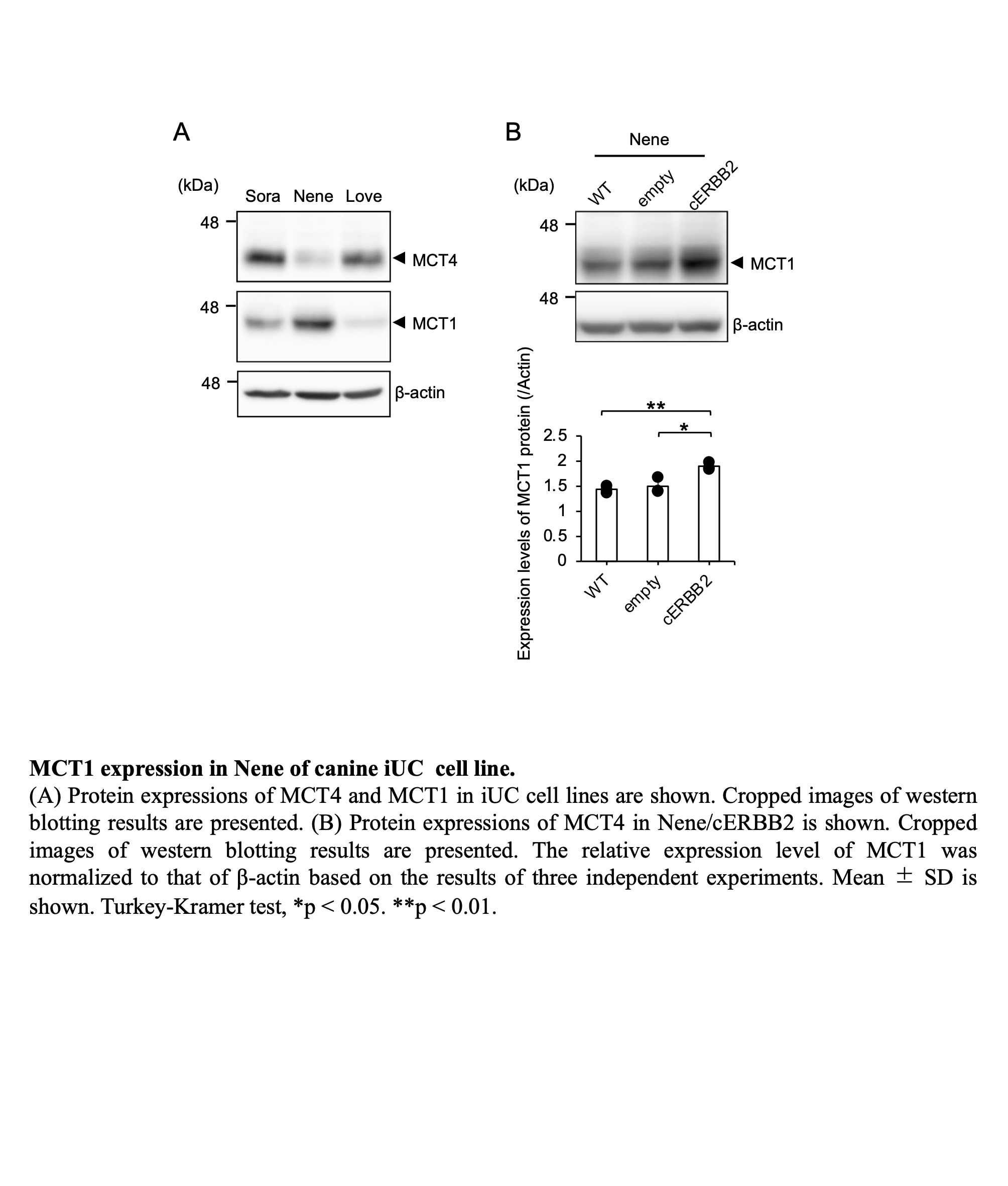

Supplement: S4 Fig — (A) Protein expressions of MCT4 and MCT1 in iUC cell lines are shown. Cropped images of western blotting results are presented. (B) Protein expressions of MCT4 in Nene/cERBB2 is shown. Cropped images of western blotting results are presented. The relative expression level of MCT1 was normalized to that of β-actin based on the results of three independent experiments. Mean ± SD is shown. Turkey-Kramer test, *p < 0.05. **p < 0.01. (TIFF) [file pone.0332825.s004.tiff]
